# Comparison of Study Controls

**DOI:** 10.1289/ehp.113-1280432

**Published:** 2005-09

**Authors:** Seiichiroh Ohsako, Chiharu Tohyama

**Affiliations:** Environmental Health Sciences Division National Institute for Environmental Studies, Tsukuba, Japan, E-mail: ohsako@nies.go.jp; Center for Disease Biology and Integrative Medicine, Graduate School of Medicine, The University of Tokyo, Tokyo, Japan, E-mail: ctohyama@m.u-tokyo.ac.jp

In the article “Natural Variability and the Influence of Concurrent Control Values on the Detection and Interpretation of Low-Dose or Weak Endocrine Toxicities,” Ashby et al. (2004) discounted a number of studies reporting low-dose effects caused by endocrine-disrupting chemicals, including bisphenol A (BPA), based on variability in control values in experiments conducted at different times. They cited data from four experiments that we reported in three published studies (Ohsako et al. 2001; Sakaue et al. 1999, 2001). Ashby et al. stated that the marked reduction in daily sperm production (DSP) caused by BPA that we observed in two experiments (Sakaue et al. 2001) should be discounted because the DSP values in BPA-exposed males were not significantly different from those in controls we reported in another study (Ohsako et al. 2001). Ashby et al. (2004) proposed that our conclusion that BPA significantly decreased DSP is incorrect because of a difference in control values for DSP from different experiments conducted at different times.

We would like to point out that it is absolutely inappropriate to compare these sets of data not only because the experiments were conducted at different times but also because they included different experimental conditions and different animals (Table 1). First, we used Sprague-Dawley rats in the two experiments in the BPA study (Sakaue et al. 2001) and Holtzman rats in another study of 2,3,7,8-tetrachlorodibenzo-*p*-dioxin (TCDD) (Ohsako et al. 2001). Second, the Sprague-Dawley rats used in the BPA study were purchased from a laboratory animal breeder, whereas the Holtzman rats used in the TCDD experiment were bred at the National Institute for Environmental Studies (NIES). Third, animal maintenance conditions were not the same for the three studies: Holtzman rats were housed in polycarbonate cages with wooden chips, and Sprague-Dawley rats were kept in hanging stainless wire-mesh cages.

There are many reasons why males from different rat strains obtained from different breeding facilities using different animal feed and housing conditions might differ in DSP. We thus disagree with the interpretation by Ashby et al. (2004) that this variability between rats from different strains invalidates the conclusion drawn by Sakaue et al. (2001) that exposure to BPA reduces DSP in Sprague-Dawley rats. We also disagree with their attempt to discount effects caused by BPA and other endocrine-disrupting chemicals due to variability in control animals from entirely different experiments in which low-dose effects were reported by other researchers.

## Figures and Tables

**Table 1:** Comparison of conditions of the three experiments.

	Sakaue et al. 1999	Ohsako et al. 2001	Sakaue et al. 2001
Rat strains	Holtzman	Holtzman	Sprague Dawley
Place of birth	Animal breeding facility (NIES)	Chemical hazard area (NIES)	CLEA Japan
Age at the time of purchase	NA	NA	PND77
Cage type	Stainless wire-mesh	Polycarbonate plastic	Stainless wire-mesh
Bedding	None	Wood chip	None
Age at the time of tissue collection	PND126	PND120	PND126
Place for examination	Animal breeding facility (NIES)	Chemical hazard area (NIES)	Animal breeding facility (NIES)
Test compound	BPA	TCDD	BPA
Vehicle	Corn oil	Corn oil/4% *n*-nonane	Corn oil/6.5% ethanol
Control DSP value^a^ (× 10^6^/testis)	39.0 ± 1.6 (4)	34.6±2.0 (12)	44.0±2.2 (5)^b^ 49.3±5.3 (8)^c^

Abbreviations: NA, not applicable; PND, postnatal day.

a Mean ± SE (number of animals).

b First experiment.

c Second experiment.
